# Quality Assessment of Socks Produced from Viscose and Lyocell Fibers

**DOI:** 10.3390/ma17071559

**Published:** 2024-03-28

**Authors:** Antoneta Tomljenović, Juro Živičnjak, Zenun Skenderi

**Affiliations:** 1 Department of Materials, Fibers and Textile Testing, University of Zagreb Faculty of Textile Technology, Prilaz baruna Filipovića 28a, 10000 Zagreb, Croatia; juro.zivicnjak@ttf.unizg.hr; 2 Department of Textile Design and Menagement, University of Zagreb Faculty of Textile Technology, Prilaz baruna Filipovića 28a, 10000 Zagreb, Croatia; zskenderi55@gmail.com

**Keywords:** socks, viscose, lyocell, yarn type, quality, textile testing

## Abstract

Most casual socks are produced from cotton and are usually combined with synthetic fibers. The suitability of viscose and lyocell fibers for knitting socks needs to be investigated further. Therefore, three series of plain socks were produced, composed in the largest content from single-spun viscose or lyocell yarns fully plated with texturized polyamide 6.6 multifilament yarn. The quality of three types of main yarns manufactured by ring, open-end rotor, and air-jet spinning processes and two types of polyamide plating yarns used in the production of socks were assessed together with the structural, usage, and comfort quality of the socks before and after simulating household laundering. In comparison with cotton socks produced from ring-spun yarns under the same conditions, the results showed that viscose and lyocell socks have better moisture absorption and breathability, comparable dimensional stability, and lower abrasion resistance; lyocell socks have lower thermal resistance; and viscose socks are less prone to surface pilling after wet pretreatment.

## 1. Introduction

Viscose fibers, conventional man-made artificial fibers made from cellulose, are produced using the indirect viscose process. Lyocell fibers are produced by regenerating cellulose in fiber form from a cellulose solution dissolved directly in an organic solvent without forming derivatives. Although both fibers are made of cellulose, they differ in their structure and properties due to differences in the production method [1].

**Figure 1:**
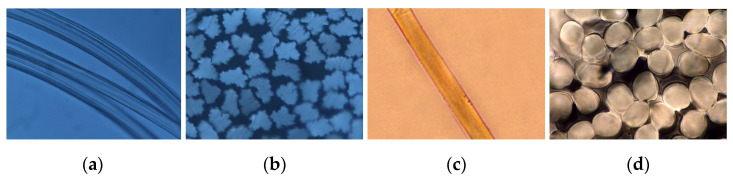
Characteristic photomicrographs of fibers (optical microscopy): (**a**) longitudinal and (**b**) cross view of viscose fibers; (**c**) longitudinal and (**d**) cross view of lyocell fibers [5].

The differences between these cellulose fibers lie primarily in their molecular structure and supramolecular arrangement, which are responsible for their mechanical and absorptive properties. The degree of polymerization, the average molecular mass, the degree of crystallinity, and the molecular orientation of lyocell fibers are significantly higher compared to viscose fibers. The ratio of crystalline to amorphous areas is approx. 9:1 for lyocell fibers, while the values for viscose fibers are approx. 6:1. Compared to other man-made cellulose fibers, this is reflected in the higher wet strength of lyocell fibers, which retain up to 85% of their dry strength. On the other hand, these parameters cause their special ability to fibrillate under the influence of external mechanical effects [2,3]. The sorption properties are also influenced by the void ratio, which is highest for viscose fibers, followed by lyocell fibers. The morphological properties of the fibers are also different—the surface and cross-section of lyocell fibers are much more regular compared to viscose (Figure 1) [1,2,3].

Knitted next-to-skin garments made of viscose or lyocell fibers, which are characterized by their silky handle, pronounced drapability and fluidity, and exceptional wearing comfort and moisture absorption, are an excellent alternative to cotton [4]. However, the suitability of viscose and lyocell fibers for knitting socks still needs to be investigated further.

Casual socks are worn inside shoes for less formal occasions and cover the foot and part of the calf. The most important parts of calf-length socks are the cuff, leg, heel, sole, toe, and foot [6,7,8]. The cuff of casual socks is usually knitted in a ribbed pattern, while the other parts are made of simple plain single jersey [6].

Most casual socks are produced from cotton and are usually combined with synthetic fibers, using polyamide or polyester as a plating yarn to improve the fit and increase the durability of the sock. Socks can also contain elastane in the structure, usually 2–3%, to improve elasticity; acrylic to reduce moisture at the interface between the sock and the skin; or polytetrafluoroethylene to reduce the coefficient of friction on the skin [9,10].

Many scientific studies have analyzed the performance of socks made of a high cotton content plated or blended with various synthetic fibers. They mostly refer to the comfort properties of the socks, e.g., comfort pressure [11,12,13], sock–skin friction [10,13], physiological and sensory parameters [13,14], thermal properties [15,16,17], moisture management, and air permeability [18,19,20], and/or to the usage properties of the socks, e.g., abrasion resistance, pilling performance [9,20,21], and dimensional stability [4,22,23,24].

A review of the literature found far fewer comparative studies on the performance of socks made from man-made cellulose artificial fibers. Modal, micro modal, viscose, bamboo, and seacell socks produced in a simple single-jersey pattern without the addition of polyamide and elastane were compared with cotton socks [25,26]. It was found that the fibers used, moisture absorption, and sock thickness influenced the comfort performance of the socks, with socks made from modal, viscose, and seacell fibers achieving the best results. The air permeability, thermal conductivity, and thermal resistance of plain single-yarn socks made from cotton, viscose, and bamboo with or without polyester and Lycra plating were also investigated [27]. Lower air permeability and thermal conductivity as well as higher thermal resistance were found in plated socks (due to their tighter and thicker knit structure). The thermal comfort of plain cotton and viscose socks in a wet state, produced with and without polyester and elastane, was also investigated [28], as well as the antimicrobial properties and wear resistance of socks made of cotton, lyocell (Tencel^LF^), and bamboo fibers plated with or without Lycra [29]. It has been found that the ribbed sock structure has better abrasion resistance and pilling properties than the plain structure and that socks made of bamboo and cotton fibers have lower abrasion resistance than socks made of lyocell [29].

Conventional ring single-spun yarns are mostly used as the main yarns for knitting socks. The fibers in ring-spun yarns are arranged in a helix, resulting in a yarn with a uniform fiber core. In unconventional open-end rotor-spun yarns, the core fibers that form the inner part of the yarn are twisted, with some fibers being wrapped. In air-jet yarns, the core fibers are arranged parallel to the yarn axis without twisting and are periodically enclosed by the wrapper fibers [30,31]. It follows that the different arrangement of fibers in yarns produced with different spinning technologies influences their properties [32]. However, the suitability of open-end rotor and air-jet-spun yarns for knitting socks still needs to be investigated further [8].

Since socks are a necessary garment, they should maintain a high level of usage and comfort quality for a lifetime. As customers today are increasingly aware of their needs and buy higher-quality products, their expectations of socks are also rising [29]. Therefore, special attention should be paid to the selection of suitable fibers and yarns for knitting socks.

The literature research has thus shown that the suitability of viscose and lyocell fibers and unconventionally spun yarns for knitting socks still needs to be sufficiently researched. A quality assessment of socks knitted with three single-spun viscose or lyocell main yarns fully plated with texturized polyamide 6.6 multifilament yarn is not found in the literature, nor are results on the effect of ring-spun cotton yarn in blends. In this paper, three series of plain single-jersey socks were therefore evaluated, with the largest content composed of single-spun viscose or lyocell yarns fully plated with texturized polyamide 6.6 multifilament yarn. The quality of three types of main yarns produced by conventional ring, unconventional open-end rotor, and air-jet spinning processes and two types of polyamide plating yarns used in the manufacture of socks were assessed. The quality of the socks was evaluated by assessing their structural, usage, and comfort quality before and after a simulated household laundering in accordance with the previously published sock evaluation methodology [8] and in comparison to cotton socks made from ring-spun yarns under the same conditions.

Accordingly, the aim of this paper was to follow up on previously published studies on the durability and comfort properties of socks made from differently spun modal and micro modal yarns [8] in order to determine the suitability of viscose and lyocell fibers for the manufacture of socks and to determine the potential synergistic effect of different yarn combinations used in socks on their quality.

## 2. Materials and Methods

### 2.1. Quality of the Yarns and the Sock Design

In this study, bright viscose (CV) and lyocell (CLY, Tencel^TM^) staple fibers with a fineness of 1.3 dtex and a length of 38/40 mm were used for the production of single-spun yarns. Three types of main yarns were spun using different spinning processes, including:

Two conventional single ring-spun yarns (Ri) made of viscose and lyocell fibers using the Saurer Zinser 351, Switzerland ring-spinning machine;

Two unconventional single open-end rotor-spun yarns (Ro) made from viscose and lyocell fibers with the Saurer Schlafhorst A8, Switzerland rotor spinning machine;

Two unconventional single air-jet-spun yarns (Ai) made from viscose and lyocell fibers with the Rieter J20, Switzerland spinning machine [32].

The fineness of the yarns used was measured in accordance with EN ISO 2060:1995 [33] and the twist of ring-spun yarns in accordance with ISO 17202:2002 [34]. The twist number of the open-end rotor-spun yarns was calculated from the rotor speed and the twist of the air-jet-spun yarns using the high air pressure in the rotating vortex of 0.6 MPa. The tensile properties of the yarns were determined in accordance with EN ISO 2062:2009 [35] using the Uster Tensorapid 4 strength tester, with a total of 100 measurements per yarn package. The unevenness of the single-spun yarns was tested in accordance with ASTM D1425/D1425M-14 [36], whereby the irregularity, the number of yarn faults, and the hairiness were determined with the Uster Tester 4-S, Switzerland at a yarn speed of 400 m/min through the capacitive measuring field and a test time of 2.5 min. The properties of the main viscose and lyocell ring-, open-end rotor-, and air-jet-spun yarns used as the main yarns for knitting socks are listed in Table 1 and Table 2.

In addition to the three single-spun yarns, one texturized multifilament polyamide plating yarn was knitted in each course of the socks for reinforcement. Two different polyamide 6.6 plating yarns were used:

PA 6.6-1, consisting of 42 monofilaments, with a fineness of 156 dtex, a tensile strength of 41.8 ± 0.5 cN/tex, and an elongation at break of 26.07 ± 0.6%;

PA 6.6-2, consisting of 68 monofilaments, with a fineness of 220 dtex, a tensile strength of 45.0 ± 0.2 cN/tex, and an elongation at break of 28.50 ± 0.2%.

An additional Lycra yarn with a fineness of 54 tex, a breaking strength of 10.2 ± 0.4 cN/tex, and an elongation at break of 321.0 ± 1.8% was added to the sock cuff [8].

**Figure 2:**
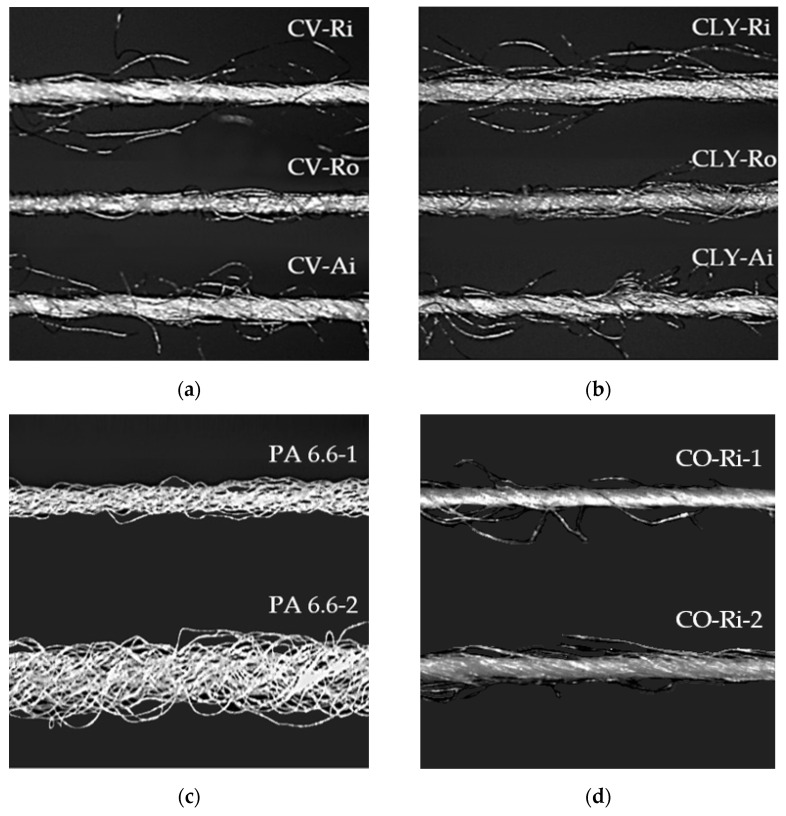
Characteristic surface structure of the yarns (optical microscopy, magnification level of 60×): (**a**) viscose ring, open-end rotor- and air-jet single-spun yarns; (**b**) lyocell ring-, open-end rotor-, and air-jet single-spun yarns; (**c**) polyamide PA 6.6-1 and PA 6.6-2 multifilament yarns; (**d**) cotton CO-Ri-1 and CO-Ri-2 ring single-spun yarns.

The quality of socks made from viscose or lyocell fibers was assessed in comparison to conventional cotton socks made from a single-spun ring yarn (CO-Ri-1) with a fineness of 20.1 ± 0.26 tex, a tensile strength of 15.1 ± 0.3 cN/tex, and an elongation at break of 3.7 ± 0.1%. Figure 2 presents optical microscopy images (taken with a Dino-Lite AM 413 ZT microscope, AnMo Electronics Corporation, Taiwan, with 60× magnification) of the single-spun main and multifilament plating yarns used in knitting.

Three series of calf-length casual socks (A, B, and C) were knitted—series A with three viscose or lyocell yarns of the same type, fully plated with PA 6.6-1 texturized yarn; series B in the same way but fully plated with PA 6.6-2 texturized yarn; and series C with PA 6.6-2, where one of the main viscose or lyocell yarns was substituted by a single-spun ring yarn (CO-Ri-2) made of cotton with a fineness of 25 tex, a tensile strength of 13.3 ± 0.3 cN/tex, and an elongation at break of 3.8 ± 0.1%. The sock design considered in this study is shown in Table 3, where the main yarns and the elastic plating yarns are indicated for each sample.

Three series of casual calf-length plain single-jersey socks in size EU 42 were produced on the Lonati Goal FL 626 sock knitting machine, Italy (gauge: E9, cylinder diameter: 95 mm (3 3/4″)). All machine settings were maintained during the knitting process. Only the cuff of the socks was knitted in a 1 × 1 ribbed pattern. The socks were stabilized on flat leg forms of a Cortese steam ironing machine after the toes had been machine sewn.

### 2.2. Quality Assessment of the Socks

In this study, the quality of the socks was evaluated by assessing their structural, usage, and comfort quality before and after five consecutive laboratory simulations of household laundry (pretreatment) in accordance with the previously published sock evaluation methodology [8].

The socks were pretreated according to EN ISO 6330:2012 [37]—washed using the gentle 3M method with Electrolux Wascator FOM71 CLS, Sweden and line-dried after each wash cycle. The washing temperature was 30 °C, and an ECE reference detergent without optical brighteners (SDC Enterprises Limited, Holmfirth, UK) was used. Before and after pretreatment, all socks were conditioned for at least 24 h on a flat surface at a temperature of 20 ± 2 °C and a relative humidity of 65 ± 4% and then sampled as required.

**Figure 3:**
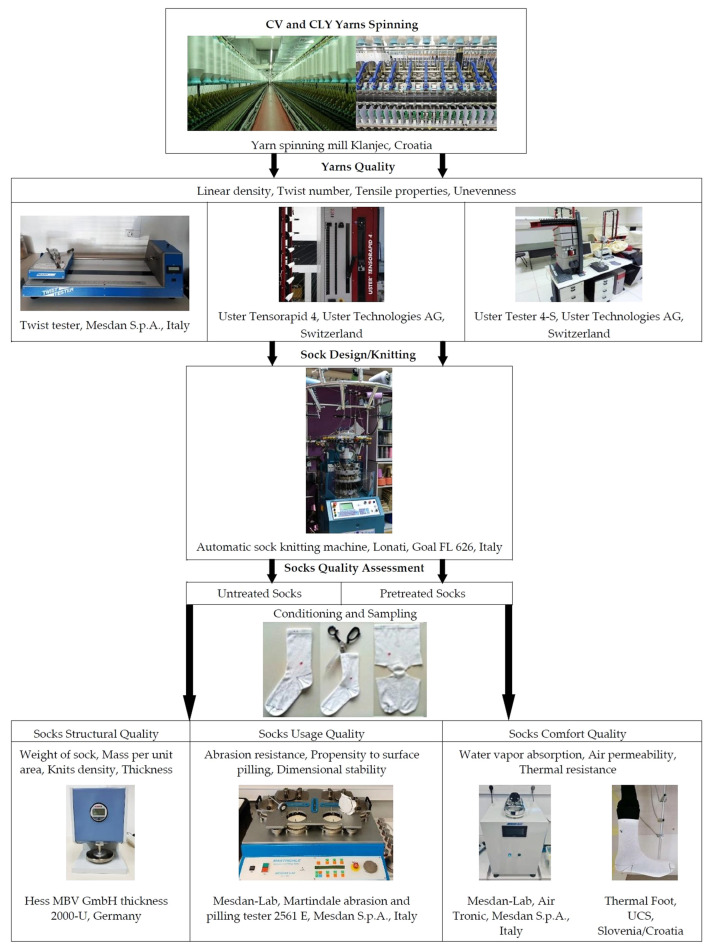
Design of the experiment.

The complete design of the experiment used in this study is shown in Figure 3.

#### 2.2.1. Structural Quality of the Socks

**Figure 4:**
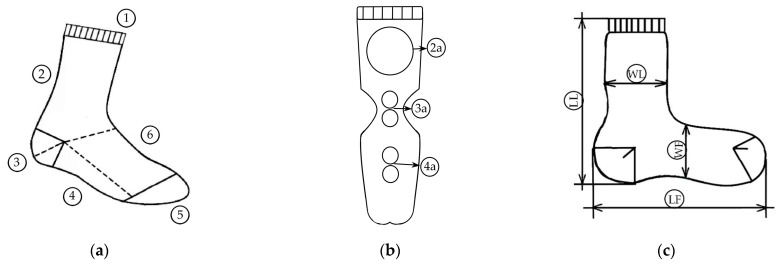
Parts of calf-length socks (**a**): (1) cuff, (2) leg, (3) heel, (4) sole, (5) toe, (6) foot [7,8]; (**b**) sampling of socks to test pilling tendency (2a) and abrasion resistance (3a,4a); (**c**) the specification for the measured sock dimensions: length and width of the leg (LL and WL), length and width of the foot (LF and WF).

The structural quality of the calf-length socks (Figure 4a) was assessed on the basis of the following properties: the weight of the sock and the mass per unit area, thickness, and density of the plain knit, determined in accordance with EN 12127:2003, EN ISO 5084:2003, and EN 14971:2008, respectively [38,39,40]. Five measurements were taken for all tests, with the exception of thickness, for which ten measurements were taken. The average result and the standard deviation were calculated.

#### 2.2.2. Usage Quality of the Socks

The usage quality of socks was assessed on the basis of abrasion resistance and the tendency to pill on the surface as well as the stability of the dimensions.

The abrasion resistance of plain knits was obtained with the Martindale abrasion apparatus according to EN 13770:2002, method 1 [41]. The specimen breakdown or significant thinning was determined, and the end point was recorded as the number of abrasion rubs. In this method, two round specimens were sampled from the heel and two from the sole area of the socks (Figure 4b), moving according to the Lissajous curve and rubbing the entire surface against a reference wool abrasive fabric (SDC Enterprises Limited, UK).

The tendency of plain knits to form surface pilling was determined using the modified Martindale method (EN ISO 12945-2:2020) [42]. Three round specimens were cut (Figure 4b) and abraded against the reference wool abrasive. After a defined number of pilling rubs, a visual assessment was carried out by three experts in accordance with EN ISO 12945-4:2020 [43], whereby ratings of 1–5 were assigned according to the degree of pilling. The average value was determined as a result.

The stability of the socks in the lengthwise and widthwise directions was measured in accordance with the proposed measurement scheme given in Figure 4c. The changes in sock dimensions, measured on the foot and leg areas after five consecutive household washes and dryings, were calculated as a percentage in accordance with EN ISO 3759:2011 and EN ISO 5077:2008 [44,45].

#### 2.2.3. Comfort Quality of the Socks

The comfort quality of the socks was assessed on the basis of the following comfort-related properties: moisture absorption, permeability to air, and thermal resistance.

The moisture absorption of plain knits was measured in accordance with ASTM D 2654-89a [46]. Three conditioned circular specimens with an area of 100 cm^2^ were weighed, then dried in an oven at 105 ± 2 °C for 24 h and weighed again. The average value of the calculated difference between the conditioned and the oven-dried mass was expressed as a percentage of moisture regain.

The air permeability of plain knits was measured in accordance with EN ISO 9237:1995 [47] with the Air Tronic Mesdan S.p.A., Italy air permeability tester using a test surface of 5 cm^2^ and a pressure drop of 100 Pa. The average of the measured air flows and the permeability to air in mm/s were recorded thereafter.

The socks thermal resistance was measured with the Thermal Foot Manikin System UCS, Slovenia/Croatia in EU size 42. The measurement was carried out under the specified ambient conditions (air temperature: 20 ± 2 °C, relative humidity: 65 ± 4%, air velocity: 1 m/s) as follows: First, the device constant (*R_ct0_*) was determined and then the total thermal resistance (*R_ctt_*) of the device and the tested sock. The calculated difference between *R_ctt_* and *R_ct0_*, expressed in m^2^ °C/W, represents the thermal resistance of the tested sock (*R_ct_*). The result is the average value of three socks from the same series.

## 3. Results and Discussion

The results contain the assessment of the structural, usage, and comfort quality of the three-sock series. The suitability of viscose and lyocell fibers for the sock manufacture and the potential synergistic effect of different yarn combinations on the quality of the socks were discussed.

### 3.1. Structural Quality Assessment of the Socks

**Figure 5:**
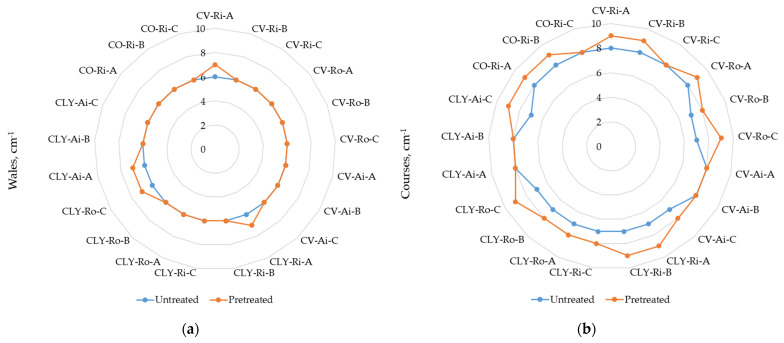
Results of the measured values of: (**a**) wale and (**b**) course density of untreated and wet pretreated sock plain knits (CV–viscose, CLY–lyocell, CO–cotton; Ri–ring spun, Ro–open-end rotor, Ai–air-jet spun; A, B, and C–sock series).

**Figure 6:**
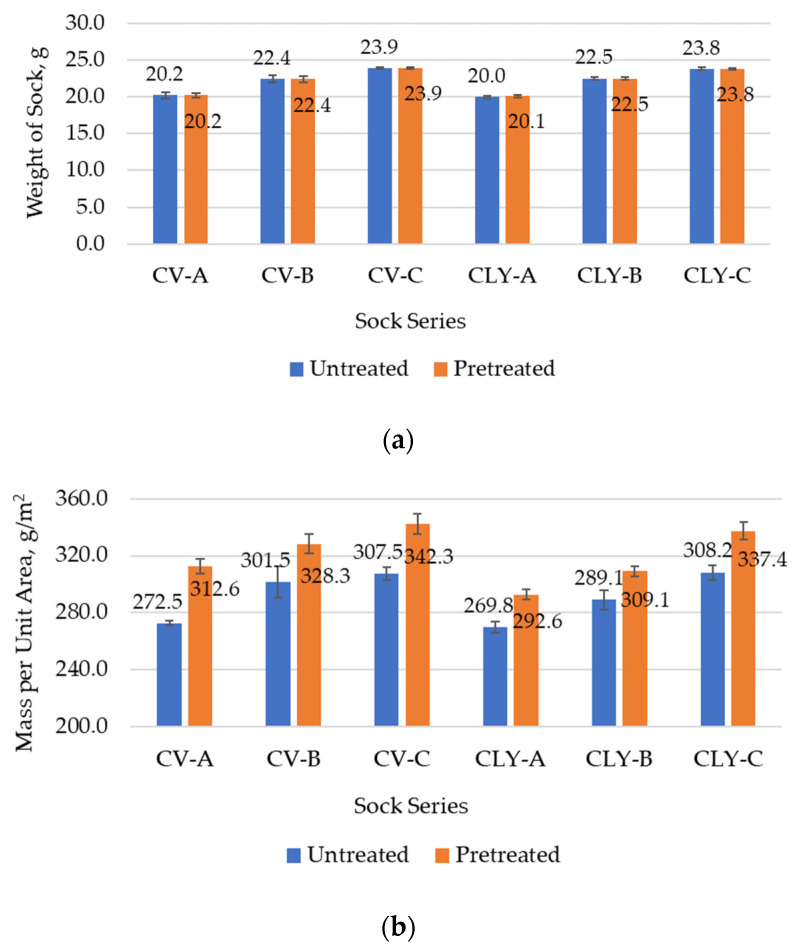

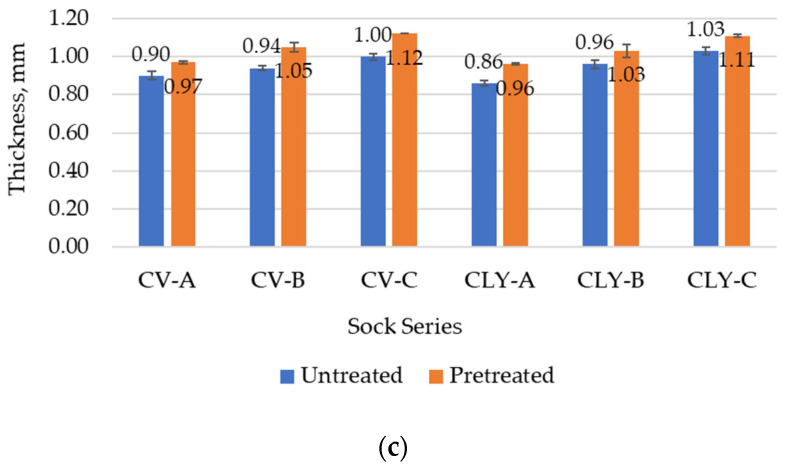
Average values of (**a**) sock weight, (**b**) mass per unit area, (**c**) and plain knit thickness determined for each sock series (A–C) produced from viscose (CV) or lyocell (CLY) fibers before and after wet pretreatment, with the corresponding standard deviation.

The measured values for the weight of the socks, the socks’ plain knit mass, the thickness, and the wale and course density, which were determined before (untreated socks) and after the five consecutive household washes and dryings of the socks (pretreated socks), are presented in Table 4 and Figure 5a,b. Figure 6 shows the average values of the structural properties that were determined for each sock series (A–C) produced from viscose or lyocell fibers.

As with the previously studied socks made from modal fibers produced in the same way (where, e.g., the average value of sock weight was 20.0 g for untreated modal series A, 22.4 g for series B, and 23.8 g for sock series C) [8], the socks of series B and C were found to have higher values for sock weight, knit mass, and thickness (Table 4), confirming that the higher-count PA 6.6 yarn used in both series and the cotton yarn in series C altered the sock mass and structure.

As expected and shown in Figure 6a, the average sock weights determined for each untreated sock series (A–C) produced from viscose or lyocell fibers were also comparable to those of the cotton socks (Table 4).

In contrast, as shown in Table 4 and Figure 6b, the results of mass/m^2^ of the sock fabric show higher values of standard deviation between measurements for each sample as well as between the same sock series produced from viscose or lyocell fibers. This can be explained by the differences observed in the wale and course density of the sock knits (Figure 5a,b) and in the yarn unevenness (Table 2). The determined number of wales/cm was the same for all untreated socks (Figure 5a), but the number of courses/cm was uneven and was usually higher for the viscose and cotton sock samples (Figure 5b), which directly led to a lower number of stitches/cm^2^ for the untreated lyocell sock samples. As shown in Table 2, the values of thin places (−50%) and thick places (+50%) measured in open-end Ro- and Ai-spun viscose (14.1 per 10^−3^ m and 57.2 per 10^−3^ m, respectively) were larger than those in lyocell (11.9 per 10^−3^ m and 49.1 per 10^−3^ m, respectively). It follows that the more uniformly structured yarns spun from lyocell fibers also had an effect on the decreased average results of mass per unit area determined for the sock series (A–C) with the highest lyocell fiber content (Figure 6b).

According to the results shown in Table 4 and Figure 6c, the thickness of the untreated socks varied only slightly. Nevertheless, somewhat higher average thickness values were determined in sock series A, produced with differently spun viscose yarns (Figure 6c), than for the same sock series produced with lyocell fibers (0.90 mm and 0.86 mm). This can be explained by the higher sock thickness values obtained for the CV-Ro-A and CV-Ai-A samples (0.90 mm and 0.93 mm, respectively), which were produced with open-end Ro- and Ai-spun viscose yarns of greater non-uniformity.

As shown in Table 4 and Figure 6a, the weight of the socks remained almost the same after five consecutive laboratory simulations of household laundry, although a minimal change of up to ±0.5% was observed in some samples. The results shown in Figure 5 for the knits’ wale and course density indicate that after wet pretreatment, the number of wales per cm increased only in the CV-Ri-A, CLY-Ri-A, CLY-Ro-C, and CLY-Ai-A socks, whereas course density increased for almost all sock samples. Due to the shrinkage of the socks, the socks become bulkier, which consequently affected the higher mass/m^2^ of viscose and lyocell sock plain knits (from 7.51% to 16.17%, and from 5.11% to 14.65%, respectively) and the thickness of viscose and lyocell sock plain knits (from 7.78% to 14.29%, and from 5.32% to 11.49%, respectively). This led us to the conclusion that the socks with the highest content of lyocell fibers have a more stable structure.

### 3.2. Usage Quality Assessment of the Socks

**Figure 7:**
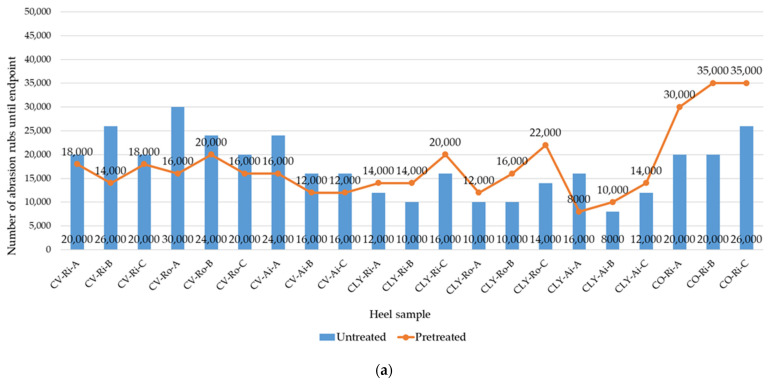

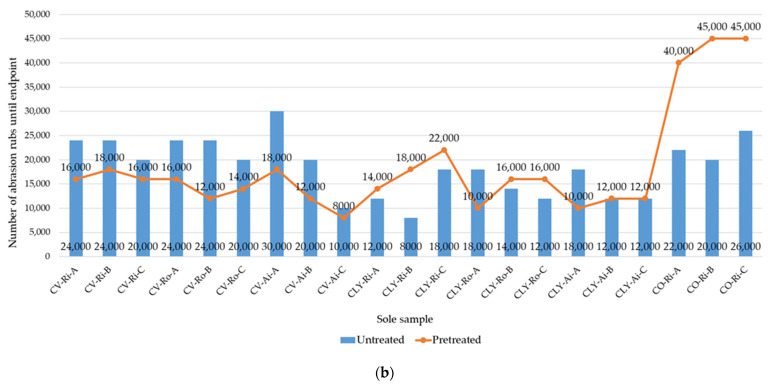
Results of the abrasion resistance of socks before and after five consecutive laboratory simulations of household laundry, determined on (**a**) the heel and (**b**) the sole (CV—viscose, CLY—lyocell, CO—cotton; Ri—ring spun, Ro—open-end rotor, Ai—air-jet spun; A, B, and C—sock series).

Socks must meet the requirements for high wear resistance [9,21]. The values of the abrasion rubs at the endpoint of the socks, measured on the heels and soles before (untreated socks) and after the five consecutive household washes and dryings (pretreated socks) using the Martindale abrasion test, are presented in Figure 7a,b.

Since polyamide filaments are considered to be very abrasion resistant [7], as in the previously tested socks made of modal fibers [8], the thinning of the spun staple yarns was determined as the endpoint for all tested socks and is presented for the socks CV-Ri-A and CLY-Ri-A in Table 5.

Although viscose fibers are considered to be the least abrasion resistant of the common textile fibers [7], in this study, untreated socks made of viscose fibers were found to rub between 16,000 and 30,000 times by the end of the test, which was comparable to cotton socks in most cases. Contrary to expectations, lower abrasion resistance values were found for socks made of lyocell, with 8000 to 18,000 rubs. Despite the fact that the lyocell yarns had better tensile properties (Table 1), the high surface fibrillation tendency of the lyocell fibers had a negative effect on the abrasion resistance of the socks. The tests also confirmed [8] that increasing the PA 6.6 count in the untreated socks of series B and C and the cotton content in the socks of series C did not improve abrasion resistance (particularly evident in the sole samples).

The abrasion resistance changed differently for all socks after the simulated household laundering (Figure 7a,b). It was found that the abrasion resistance of socks made of viscose fibers was up to 60% lower after wet pretreatment, while it was generally up to 63% higher for socks made of lyocell fibers (Table 5) and 56% higher for socks made of cotton. One of the parameters influencing abrasion is the tensile strength of the fibers, which is lowest for viscose fibers when wet [2]. Therefore, the plain knits of socks made of cotton and lyocell fibers showed increased abrasion resistance after wet pretreatment. Considering comprehensively, the wear quality of socks made of viscose and lyocell fibers after wet pretreatment is very similar but significantly lower than that of socks made of cotton, where the endpoint is 30,000–45,000 rubs. Tests on pretreated socks have also shown that an increased proportion of elastic PA 6.6 yarn in series B and C lyocell socks improves their abrasion resistance.

In contrast to the same socks made from modal fibers [8], where, e.g., the untreated sock knits made from ring-spun yarns showed better abrasion resistance, no significant influence of the main yarn type spun with different spinning methods (ring, open-end rotor, or air-jet) on the abrasion resistance results achieved was found in this study.

During wear, the pills become entangled on the knit surface, which leads to a reduction in the esthetic and usage quality of the socks [9,21]. In this study, as expected, the tendency of plain knits to form surface piles, assessed before and after household laundering, was higher after prolonged wear simulation (Table 6).

Untreated series A lyocell and viscose socks were less susceptible to surface pilling, especially those made from air-jet and open-end rotor-spun yarns, with lower hairiness compared to ring-spun yarns (Table 2 and Figure 2). At the same time, after being rubbed 7000 times, the lyocell socks were assessed with excellent grades of 4 and 4/5 at the end of the test, whereas the viscose samples were rated with slightly lower ratings of 4 and 3/4 compared to the cotton socks, which received lower grades.

As with the previously tested socks made of modal fibers [8], it was confirmed that increasing the PA 6.6 count in the untreated socks of series B and C and the cotton content in the socks of series C did not improve their surface esthetic properties and results in lower grades.

After rubbing 7000 times, a higher tendency to form surface pilling was observed in all tested socks at the end of the test (Table 6). The best pilling values after the five consecutive pretreatment cycles were achieved with viscose socks made from yarns produced using air-jet spinning technology. These socks of series A and B showed moderate pilling (rated as grade 2 and 2/3) compared to cotton socks and socks made of lyocell fibers, with a higher tendency to surface fibrillation.

The calculated percentage change in the measurements of the leg and foot lengths as well as the leg and foot widths of the socks after five consecutive household washes and dryings is shown in Table 7.

The dimensional changes were more pronounced in the longitudinal direction of the socks (Table 7) and could be connected with more intensive changes in courses density of the wet pretreated socks (Figure 5a,b).

The determined shrinkage in the longitudinal direction of the leg part of the viscose socks was between 0.0% and 8.3%, whereby the highest value was determined for the sock sample CV-Ri-C, and for the lyocell socks between 0.0% and 10.6%, whereby the highest value was determined for the sock CLY-Ai-A. The shrinkage in foot length was higher on average for the viscose socks, ranging between 4.1% and 8.0%, and between 3.9% and 12.7% for the lyocell socks.

The measured width of the sock leg remained unchanged in almost all socks after pretreatment, with the exception of the socks in series A, which were made from ring-spun yarns (CV-Ri-A, CLY-Ri-A, and CO-Ri-A). The foot width of the various heavier viscose and lyocell socks in groups B and/or C also remained unchanged after pretreatment (Table 7). The decrease in polyamide fineness primarily reduced the shrinkage in the transverse direction of series B and C socks after household care, as in previous tests [8].

In general, for most of the tested samples with the highest viscose and lyocell fiber content, the determined shrinkage was lower or comparable to the same dimensional changes determined for conventional cotton socks.

### 3.3. Comfort Quality Assessment of the Socks

**Figure 8:**
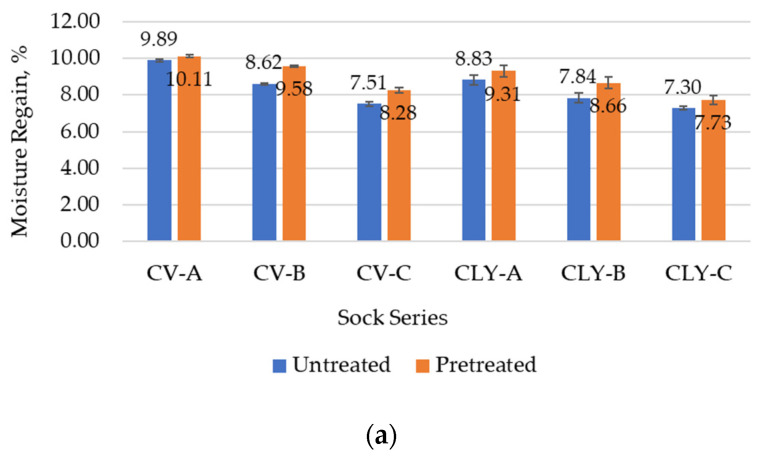

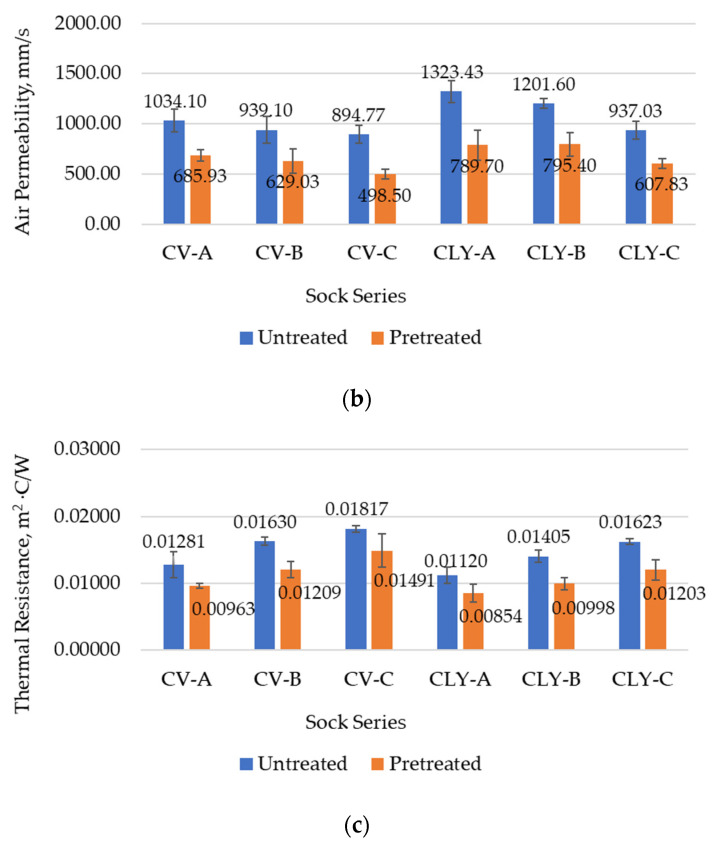
Average values of (**a**) moisture regain and (**b**) air permeability of sock plain knits; (**c**) thermal resistance of socks determined for each sock series (A–C) produced from viscose (CV) or lyocell (CLY) fibers before and after wet pretreatment, with the corresponding standard deviation.

Moisture absorption, air flow, and heat transport through the socks largely determine the quality of their wearing comfort [8,16]. The measured values for the moisture absorption and air permeability of the socks as well as the thermal resistance, which were carried out before (untreated socks) and after the five consecutive household washes and dryings of the socks (pretreated socks), are therefore listed in Table 8. Figure 8 shows the average values of the comfort-related properties determined for each sock series (A–C) produced from viscose or lyocell fibers.

The results of moisture regain shown in Table 8 indicate that the hydrophilicity decreased in the socks of series B and C with a more compact structure. The average value of moisture regain for untreated viscose series A was 9.89%, for series B was 8.62%, and for sock series C was 7.51% (Figure 8a), whereas the values for lyocell sock series (A–C) were 8.83%, 7.84%, and 7.30%, respectively, whereby the highest results were found for series A and B socks made from yarns produced with air-jet technology.

Since the viscose sock samples showed better moisture absorption than lyocell and cotton socks, it follows that the observed differences are primarily related to fiber type, hydrophilicity, and morphology [1,2,3]. In support of this assumption, as in the earlier study with modal socks (where the average value of moisture absorption was 9.28% for the untreated modal series A, 8.22% for series B, and 7.02% for sock series C) [8], moisture absorption values were found to decrease with a higher proportion of cotton and polyamide, which have lower hydrophilicity than the cellulose man-made fibers used in sock series B and C.

After the wet pretreatment, hydrophilicity increased due to full relaxation of all socks (Table 8). The moisture regain of the pretreated socks increased by 15% for the viscose socks, 13% for the lyocell socks, and 20% for the conventional cotton socks. The highest increase was observed in the sock samples of series B (Table 8 and Figure 8a), which had a better ability to absorb moisture.

The higher air flow ensures the breathability of the socks and was primarily influenced by the thickness of the fabric and the open porosity [19,48]. As shown in Table 8, permeability to air decreased in all socks with a more compact structure (series B and C), whereas untreated socks made of lyocell fibers were more breathable (Table 8 and Figure 8b). This can be explained by the smooth cross-section and smooth surface of the lyocell fibers, which facilitate the passage of air through the fabric.

The sock fabric air permeability results show higher standard deviation values between measurements for each sample (Table 8) as well as between the same sock series produced from lyocell or viscose fibers (Figure 8b). The average value of air permeability for viscose series A was 1034.10 mm/s, for series B was 939.10 mm/s, and for sock series C was 894.77 mm/s (Figure 8b), whereby the highest results were found for socks made from yarns produced with the open-end rotor system and the lowest for those made from yarns produced with the conventional ring system (Table 8). For the socks produced from lyocell fibers, the average air permeability values were 1323.43 mm/s, 1201.60 mm/s, and 937.03 mm/s for series A, B, and C, respectively, with the highest results also found in the socks made of yarns produced with the open-end rotor technology and the lowest values in the socks produced with the conventional ring system as well as with the unconventional air-jet system.

It was found that the measured irregularity and the number of thin and thick places and neps in the main single-spun yarns had a significant influence on the results obtained. Although moderate values for hairiness were obtained for the open-end rotor-spun yarns made from viscose and lyocell fibers, these had the highest values for overall unevenness (Table 2), which primarily affected the results for increased airflow obtained for viscose and lyocell socks produced from open-end rotor-spun yarns (Table 8).

After the household laundry simulation, the airflow through the sock structure was reduced due to changes in its dimensions (Table 8 and Figure 8b). The permeability of the wet pretreated socks decreased by 52% for the viscose socks, 50% for the lyocell socks, and 44% for the conventional socks made from cotton.

The results of the average thermal resistance shown in Figure 8c indicate that resistance to heat flow in the socks of series B and C increased with increasing thickness and more compact structure (Table 4) and thus the volume of trapped air [49] that prevents heat transfer. For all sock series (A–C), it was found that the socks made of lyocell fibers had the lowest thermal resistance values before and after wet pretreatment.

However, as shown in Table 8, the thermal resistance of socks varies depending on the type of spun yarn used in their manufacture. The untreated socks of all series (A–C), which were knitted with viscose yarns produced with the conventional ring system, had a higher resistance to heat flow (0.01504 m^2^·C/W, 0.01706 m^2^·C/W, and 0.01866 m^2^·C/W, respectively) than the socks produced with air-jet (0.01309 m^2^·C/W, 0.01620 m^2^·C/W, and 0.01835 m^2^·C/W, respectively) or open-end rotor yarns (0.01030 m^2^·C/W, 0.01563 m^2^·C/W, and 0.01749 m^2^·C/W, respectively). The finding that the irregularity was lowest in the viscose yarns produced by the ring spinning process, which follow yarns produced by the air-jet and open-end rotor processes (11.50%, 13.31%, and 14.63%, respectively, as shown in Table 2), indicates that the single-spun yarns with less irregularity and the knitting structure produced from them had a great influence on the results obtained. It can therefore be concluded that viscose socks made from less irregular yarns are less permeable to air and more resistant to heat.

In addition, it is noted that for all socks series produced from lyocell fibers before wet pretreatment, the highest heat resistance was obtained for sock structures knitted with yarns produced with the air-jet spinning system, which had the lowest irregularity (11.93%) and hairiness (3.53), and lower heat resistance was obtained for socks knitted with ring-spun and open-end rotor-spun yarns, which had higher irregularity (12.21% and 14.77% respectively) and hairiness (6.50 and 4.73, respectively). The resistance to heat transfer through the knitted structure of cotton socks was comparable to that of viscose socks made from conventional ring-spun yarns and greater than that of the same lyocell socks.

The shrinkage of the pretreated socks (Table 7) increased the porosity of the sock structure when measured stretched on the thermal foot and primarily affected the lower thermal resistance of all tested socks after the five consecutive household washes and dryings (Table 8 and Figure 8c).

## 4. Conclusions

The suitability of viscose and lyocell fibers for sock manufacture and the potential synergistic effect of different yarn combinations on the quality of socks were discussed. The three series of viscose and lyocell socks designed were assessed based on the basis of their structural, usage, and comfort quality before and after five consecutive laboratory simulations of household laundry. The results show that the socks differed in quality. The following conclusions can be drawn from this.

Socks with the highest content of lyocell fibers, with a smooth cross-section and smooth surface and a more even structure of the lyocell single-spun yarns made from them had the most stable knitted structure. Despite the fact that the lyocell yarns had better tensile properties, the high surface fibrillation tendency of the lyocell fibers negatively affected the abrasion resistance of the socks, for which lower values were determined. It varied in all the socks tested after the wet pretreatment—it decreased by up to 60% in the viscose socks and increased by up to 63% in the lyocell socks and by up to 56% in the cotton socks. The wear quality of CV and CLY socks was found to be very similar after pretretment but significantly lower than that of socks made of cotton and modal fibers [8], where the endpoint was 3000–45,000 rubs. However, in contrast to the same socks made of modal fibers [8], no significant influence of the main yarn type spun with different spinning methods on the abrasion resistance results obtained was found in this study. Compared to cotton socks, lyocell socks were less susceptible to surface pilling, especially those made from air-jet and open-end rotor-spun yarns, where only partial pills (grade 4 and 4/5) formed after 7000 pilling rubs. After the five consecutive pretreatment cycles, lower final grades were observed, with better pilling values being achieved in viscose socks made from yarns produced using air-jet spinning technology. The dimensional change was more pronounced in the longitudinal direction of the socks and amounted to a maximum of 10.6% and 12.7% for CV and CLY socks, respectively, i.e., less than for socks made of modal fibers [8]. The decrease in polyamide fineness mainly reduces the socks shrinkage in the transverse direction after household care.

It can be concluded that viscose and lyocell socks have a comparable dimensional stability and lower abrasion resistance compared to cotton socks, and that viscose socks are less susceptible to surface pilling after wet pretreatment.

Moisture regain decreases with thicker and heavier socks, with viscose socks showing higher values than lyocell, modal [8], and cotton socks. After the wet pretreatment, the moisture absorption increased due to full relaxation of the socks by up to 15% for CV socks, up to 13% for CLY socks, and up to 20% for conventional cotton socks. Air permeability decreased in socks with a more compact structure, while untreated socks made of lyocell fibers were more breathable and comparable to modal socks [8]. The overall unevenness of the open-end rotor-spun yarns was found to primarily influence the higher air permeability values obtained for viscose and lyocell socks. After the wet pretreatment, the air flow through the sock structure was reduced by up to 52% for CV socks, by up to 50% for CLY socks, and by up to 44% for cotton socks due to the altered dimensions. Larger thermal resistance was determined for more compact structures, which contain a larger volume of trapped air that prevents heat transfer, such as socks made of modal microfibers [8] and viscose fibers. Lyocell socks offer lower resistance to heat, which suggests that the more uniform structure of the lyocell yarns produced using air-jet spinning technology and the knitting structure had a major influence on the results achieved. The shrinkage of the pretreated socks increased the porosity of the sock structure when measured stretched on the thermal foot and primarily affected the lower thermal resistance of all the socks.

From this, one could conclude that viscose and lyocell socks have better moisture absorption and breathability compared to cotton socks and that lyocell socks have lower thermal resistance.

The test results obtained will hopefully be useful in the development of socks made from a blend of viscose and lyocell fibers. As the research continues, various innovative blends with cotton and differently structured plating yarns will be evaluated for knitting socks.

## Figures and Tables

**Table 1:** Fineness, twist, and tensile properties of the main single-spun viscose and lyocell yarns.

Main Yarn *	Fineness, Tex	Twist, m^−1^	Tensile Strength, cN/tex	Breaking Elongation, %	Work of Rupture, Ncm
CV-Ri	19.9 ± 0.13	751 ± 12.00	16.80 ± 1.26	14.41 ± 1.01	14.81 ± 1.81
CV-Ro	20.1 ± 0.15	753 **	13.98 ± 0.26	11.23 ± 1.06	10.29 ± 1.62
CV-Ai	20.2 ± 0.14	- ***	14.52 ± 1.42	9.58 ± 1.48	9.12 ± 2.06
CLY-Ri	20.1 ± 0.28	810 ± 14.60	26.86 ± 2.02	9.96 ± 0.70	15.65 ± 2.12
CLY-Ro	19.8 ± 0.32	753 **	19.07 ± 2.11	8.37 ± 0.81	9.50 ± 1.78
CLY- Ai	20.2 ± 0.30	- ***	25.26 ± 1.88	9.25 ± 0.75	13.22 ± 1.97

* CV—viscose, CLY—lyocell; Ri—ring spun, Ro—open-end rotor spun, Ai—air-jet spun; ** rotor speed, *** air pressure 0.6 MPa.

**Table 2:** Unevenness of the main single-spun viscose and lyocell yarns.

Main Yarn *	Irregularity (CVm), %	Thin Places (−50%), 10^−3^ m	Thick Places (+50%), 10^−3^ m	Neps (+200%), 10^−3^ m	Hairiness
CV-Ri	11.50 ± 0.18	0	10.3 ± 4.30	34.7 ± 10.13	6.47 ± 0.25
CV-Ro	14.63 ± 0.10	14.1 ± 7.60	57.2 ± 11.20	102.5 ± 12.81	4.36 ± 0.08
CV-Ai	13.31 ± 0.37	7.9 ± 4.10	10.1 ± 6.70	10.4 ± 3.21	3.75 ± 0.21
CLY-Ri	12.21 ± 0.10	0	10.9 ± 3.98	44.15 ± 13.25	6.50 ± 0.21
CLY-Ro	14.77 ± 0.31	11.9 ± 6.50	49.1 ± 13.70	141.9 ± 60.02	4.73 ± 0.14
CLY- Ai	11.93 ± 0.20	0.5 ± 0.76	3.6 ± 1.98	18.4 ± 5.32	3.53 ± 0.05

* CV—viscose, CLY—lyocell; Ri—ring spun, Ro—open-end rotor spun, Ai—air-jet spun.

**Table 3:** Sock design.

Sock Series	Yarns Used	
A	Main yarns	Viscose: ring, open-end rotor, or air-jet single-spun × 3 Lyocell: ring, open-end rotor, or air-jet single-spun × 3 Cotton: ring single-spun-1 × 3
	Plating yarns	Texturized multifilament polyamide 6.6-1 × 1 Single Lycra × 1 (in the cuff)
B	Main yarns	Viscose: ring, open-end rotor, or air-jet single-spun × 3 Lyocell: ring, open-end rotor, or air-jet single-spun × 3 Cotton: ring single-spun-1 × 3
	Plating yarns	Texturized multifilament polyamide 6.6-2 × 1 Single Lycra × 1 (in the cuff)
C	Main yarns	Viscose: ring, open-end rotor, or air-jet single-spun × 2 Lyocell: ring, open-end rotor, or air-jet single-spun × 2 Cotton: ring single-spun-1 × 2
		+Cotton: ring single-spun-2 × 1
	Plating yarns	Texturized multifilament polyamide 6.6-2 × 1 Single Lycra × 1 (in the cuff)

**Table 4:** Structure-related properties obtained for the untreated and wet pretreated socks, with the corresponding standard deviation.

Sock *	Sock Weight, g		Sock Knit Mass per Unit Area, g/m^2^		Sock Knit Thickness, mm	
	Untreated	Pretreated	Untreated	Pretreated	Untreated	Pretreated
CV-Ri-A	19.7 ± 0.01	19.8 ± 0.01	274.6 ± 1.51	305 ± 1.87	0.88 ± 0.05	0.96 ± 0.04
CV-Ri-B	21.7 ± 0.10	21.8 ± 0.09	312.0 ± 1.95	335 ± 2.25	0.93 ± 0.02	1.04 ± 0.02
CV-Ri-C	23.7 ± 0.02	23.7 ± 0.03	301.6 ± 1.82	350 ± 2.45	0.99 ± 0.03	1.12 ± 0.02
CV-Ro-A	20.7 ± 0.08	20.7 ± 0.07	272.2 ± 1.48	318 ± 2.02	0.90 ± 0.04	0.97 ± 0.04
CV-Ro-B	22.9± 0.03	22.9 ± 0.05	306.2 ± 1.88	331 ± 2.19	0.94 ± 0.01	1.03 ± 0.02
CV-Ro-C	24.1 ± 0.02	24.1 ± 0.03	311.9 ± 1.95	343 ± 2.36	0.98 ± 0.02	1.12 ± 0.01
CV-Ai-A	20.3 ± 0.10	20.2 ± 0.09	270.8 ± 1.47	315 ± 1.98	0.93 ± 0.04	0.98 ± 0.02
CV-Ai-B	22.5 ± 0.03	22.6 ± 0.03	286.4 ± 1.64	319 ± 2.04	0.96 ± 0.03	1.09 ± 0.03
CV-Ai-C	24.0 ± 0.08	23.9 ± 0.07	309.1 ± 1.91	333 ± 2.22	1.02 ± 0.02	1.12 ± 0.02
CLY-Ri-A	19.8 ± 0.02	19.9 ± 0.02	274.5 ± 1.51	296 ± 1.75	0.86 ± 0.05	0.98 ± 0.04
CLY-Ri-B	22.2 ± 0.03	22.3 ± 0.03	298.6 ± 1.78	314 ± 1.97	0.93 ± 0.02	1.05 ± 0.02
CLY-Ri-C	23.5 ± 0.09	23.6 ± 0.08	314.2 ± 1.97	334 ± 2.23	1.02 ± 0.03	1.10 ± 0.02
CLY-Ro-A	20.1 ± 0.02	20.1 ± 0.04	264.4 ± 1.40	288 ± 1.66	0.87 ± 0.03	0.97 ± 0.03
CLY-Ro-B	22.5 ± 0.03	22.6 ± 0.03	283.2 ± 1.60	307 ± 1.89	0.96 ± 0.03	1.03 ± 0.03
CLY-Ro-C	24.0 ± 0.10	23.9 ± 0.08	308.7 ± 1.91	332 ± 2.21	1.00 ± 0.03	1.10 ± 0.01
CLY-Ai-A	20.3 ± 0.08	20.4 ± 0.04	270.6 ± 1.46	294 ± 1.73	0.87 ± 0.02	0.96 ± 0.02
CLY-Ai-B	22.8 ± 0.03	22.7 ± 0.04	285.5 ± 1.63	306 ± 1.88	0.99 ± 0.03	1.07 ± 0.02
CLY-Ai-C	23.8 ± 0.09	23.8 ± 0.10	301.6 ± 1.82	346 ± 2.39	1.05 ± 0.02	1.12 ± 0.03
CO-Ri-A	20.0 ± 0.04	20.0 ± 0.04	271.8 ± 1.48	289 ± 1.67	0.84 ± 0.04	0.96 ± 0.02
CO-Ri-B	22.7 ± 0.05	22.6 ± 0.06	289.2 ± 1.67	314 ± 1.98	0.94 ± 0.04	0.99 ± 0.02
CO-Ri-C	23.8 ± 0.09	23.8 ± 0.08	308.1 ± 1.90	326 ± 2.13	1.03 ± 0.03	1.11 ± 0.02

* CV—viscose, CLY—lyocell, CO—cotton, Ri—ring spun, Ro—open-end rotor spun, Ai—air-jet spun; A, B, and C—sock series.

**Table 5:** Surface condition of the sole knits CV-Ri-A and CLY-Ri-A, before and after 10,000 abrasion rubs and after reaching significant thinning.

Sock *	**CV-Ri-A**		
Untreated	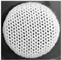	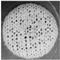	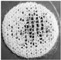
Number of Rubs	0	10,000	24,000
Pretreated	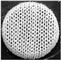	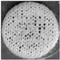	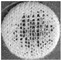
Number of Rubs	0	10,000	16,000
Sock *	**CLY-Ri-A**		
Untreated	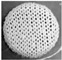	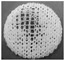	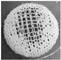
Number of Rubs	0	10,000	12,000
Pretreated	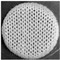	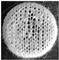	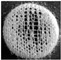
Number of Rubs	0	10,000	14,000

* CV—viscose, CLY—lyocell; Ri—ring spun; A—sock series.

**Table 6:** Degree of pilling on the visually assessed surface of socks after an appropriate number of pilling rubs.

Sock *	Untreated						Pretreated					
		No. of Rubs										
	125	500	1000	2000	5000	7000	125	500	1000	2000	5000	7000
					Pilling Grades							
CV-Ri-A	4/5	4	4	3/4	3	3	4	3	3	3	2	1
CV-Ri-B	5	5	4/5	4/5	4	3/4	5	4	3/4	3	2	1/2
CV-Ri-C	5	4/5	4	3/4	3	2/3	4/5	4	3	2/3	1	/
CV-Ro-A	4/5	4/5	4	3/4	3/4	3/4	4	4	3	2	2	1
CV-Ro-B	5	4/5	4/5	4	3/4	3	5	4/5	4	3/4	2/3	2
CV-Ro-C	5	4/5	4	3	2/3	2	4/5	4	3	2	1	/
CV-Ai-A	4/5	4/5	4/5	4	4	4	5	5	4	3	3	2/3
CV-Ai-B	5	5	4/5	4/5	4/5	3/4	5	4/5	4/5	4	3	2
CV-Ai-C	5	4/5	4	3/4	3	2/3	4/5	4	3	2/3	1	/
CLY-Ri-A	4/5	4	4	4	4	3/4	5	4	4	3	2	1
CLY-Ri-B	5	5	4	3/4	3/4	2/3	4	3/4	3	3	2/3	1
CLY-Ri-C	5	4/5	4	3/4	3	2/3	4/5	4	3	2/3	1	/
CLY-Ro-A	5	5	5	5	5	4/5	5	4	3/4	3	2	1
CLY-Ro-B	5	4/5	4/5	4	4	3/4	4	3/4	3	2/3	1/2	1
CLY-Ro-C	5	4/5	4	3/4	3	2/3	4/5	4	3	2/3	1	/
CLY-Ai-A	5	4/5	4/5	4	4	4	5	4	3/4	3	2	1
CLY-Ai-B	5	5	5	4/5	4	3/4	4/5	4	3/4	3	2/3	2
CLY-Ai-C	5	4/5	4	3/4	3	2/3	4/5	4	3	2	1	/
CO-Ri-A	4/5	4	3/4	3	3	2/3	5	4	3	2/3	1/2	1
CO-Ri-B	4/5	4	4	3/4	3	2/3	4/5	4	3	2/3	2	1/2
CO-Ri-C	4/5	4	3/4	3	3	2	4/5	4	3	3	2	1

* CV—viscose, CLY—lyocell, CO-cotton; Ri—ring spun, Ro—open-end rotor spun, Ai—air-jet spun; A, B, and C—sock series.

**Table 7:** Percentage change in sock dimensions lengthwise and crosswise after five consecutive household washes and dryings.

Sock *	Dimensional Stability, %			
	Length of the Leg	Length of the Foot	Width of the Leg	Width of the Foot
CV-Ri-A	0.0	−8.0	−5.9	−5.6
CV-Ri-B	−2.3	−8.0	0.0	0.0
CV-Ri-C	−8.3	−6.0	0.0	0.0
CV-Ro-A	−2.2	−6.0	0.0	−5.6
CV-Ro-B	−2.2	−7.8	0.0	0.0
CV-Ro-C	0.0	−6.0	0.0	0.0
CV-Ai-A	−2.2	−4.1	0.0	−5.6
CV-Ai-B	−2.2	−7.8	0.0	0.0
CV-Ai-C	−6.3	−7.8	0.0	−5.3
CLY-Ri-A	−4.3	−5.9	−5.9	−11.1
CLY-Ri-B	0.0	−5.8	0.0	−5.6
CLY-Ri-C	−4.2	−5.9	0.0	0.0
CLY-Ro-A	−4.3	−5.9	0.0	−5.6
CLY-Ro-B	0.0	−5.9	0.0	0.0
CLY-Ro-C	−6.4	−12.7	0.0	−5.3
CLY-Ai-A	−10.6	−3.9	0.0	−5.6
CLY-Ai-B	−6.4	−11.1	0.0	−5.3
CLY-Ai-C	−4.3	−4.0	0.0	−5.3
CO-Ri-A	−4.3	−7.7	−5.3	0.0
CO-Ri-B	−4.3	−7.7	0.0	−5.3
CO-Ri-C	−6.1	−7.7	0.0	0.0

* CV—viscose, CLY—lyocell, CO-cotton; Ri—ring spun, Ro—open-end rotor spun, Ai—air-jet spun; A, B, and C—sock series.

**Table 8:** Comfort-related properties obtained for untreated and wet pretreated socks, with the corresponding standard deviation.

Sock *	Sock Knit Moisture Regain, %		Sock Knit Air Permeability, mm/s		Sock Thermal Resistance, m^2^·C/W	
	Untreated	Pretreated	Untreated	Pretreated	Untreated	Pretreated
CV-Ri-A	9.94 ± 0.01	10.01 ± 0.04	890.20 ± 94.18	744.30 ± 37.44	0.01504 ± 0.00178	0.00986 ± 0.00116
CV-Ri-B	8.59 ± 0.03	9.66 ± 0.01	794.30 ± 65.85	524.90 ± 77.32	0.01706 ± 0.00284	0.01129 ± 0.00242
CV-Ri-C	7.53 ± 0.01	8.14 ± 0.03	785.60 ± 144.55	544.70 ± 119.73	0.01866 ± 0.00145	0.01639 ± 0.00142
CV-Ro-A	9.80 ± 0.06	10.18 ± 0.05	1174.30 ± 84.90	706.90 ± 74.79	0.01030 ± 0.00138	0.00904 ± 0.00117
CV-Ro-B	8.58 ± 0.04	9.56 ± 0.04	1118.80 ± 164.58	801.70 ± 135.41	0.01563 ± 0.00162	0.01384 ± 0.00155
CV-Ro-C	7.38 ± 0.01	8.48 ± 0.06	1002.70 ± 104.88	520.50 ± 175.3	0.01749 ± 0.00190	0.01688 ± 0.00177
CV-Ai-A	9.94 ± 0.05	10.14 ± 0.01	1037.80 ± 159.20	606.60 ± 172.15	0.01309 ± 0.00191	0.00997 ± 0.00287
CV-Ai-B	8.70 ± 0.01	9.53 ± 0.02	904.20 ± 113.21	560.50 ± 72.81	0.01620 ± 0.00179	0.01113 ± 0.00265
CV-Ai-C	7.61 ± 0.03	8.22 ± 0.01	896.00 ± 59.94	430.30 ± 62.35	0.01835 ± 0.00177	0.01146 ± 0.00199
CLY-Ri-A	8.63 ± 0.01	9.16 ± 0.01	1232.10 ± 136.39	616.10 ± 188.22	0.00968 ± 0.00265	0.00666 ± 0.00181
CLY-Ri-B	7.67 ± 0.04	8.66 ± 0.05	1133.30 ± 132.26	640.10 ± 153.88	0.01283 ± 0.00103	0.00880 ± 0.00233
CLY-Ri-C	7.20 ± 0.01	7.59 ± 0.02	927.30 ± 132.14	616.40 ± 58.06	0.01640 ± 0.00123	0.01075 ± 0.00266
CLY-Ro-A	8.66 ± 0.06	9.04 ± 0.06	1476.90 ± 127.60	981.00 ± 246.62	0.01118 ± 0.00182	0.00973 ± 0.00181
CLY-Ro-B	7.61 ± 0.04	8.28 ± 0.04	1234.90 ± 171.28	932.00 ± 140.83	0.01443 ± 0.00115	0.01094 ± 0.00128
CLY-Ro-C	7.44 ± 0.04	7.51 ± 0.05	1047.00 ± 95.17	664.10 ± 44.96	0.01568 ± 0.00181	0.01112 ± 0.00133
CLY-Ai-A	9.20 ± 0.01	9.72 ± 0.01	1261.30 ± 93.59	772.00 ± 57.36	0.01273 ± 0.00203	0.00922 ± 0.00247
CLY-Ai-B	8.24 ± 0.02	9.05 ± 0.03	1236.60 ± 170.90	814.10 ± 174.38	0.01488 ± 0.00094	0.01021 ± 0.00273
CLY-Ai-C	7.26 ± 0.01	8.08 ± 0.05	836.80 ± 94.31	543.00 ± 45.18	0.01660 ± 0.00175	0.01421 ± 0.00235
CO-Ri-A	5.91 ± 0.01	6.35 ± 0.02	839.80 ± 86.42	511.10 ± 40.94	0.01674 ± 0.00377	0.00831 ± 0.00069
CO-Ri-B	5.22 ± 0.03	6.27 ± 0.01	770.40 ± 78.73	475.10 ± 82.14	0.01700 ± 0.00038	0.00960 ± 0.00083
CO-Ri-C	4.68 ± 0.01	5.01 ± 0.03	712.70 ± 55.95	399.70 ± 69.39	0.017600± 0.00159	0.01600 ± 0.00136

* CV—viscose, CLY—lyocell, CO-cotton; Ri—ring spun, Ro—open-end rotor spun, Ai—air-jet spun; A, B, and C—sock series.

## Data Availability

Data are available in a publicly accessible repository.
